# Influence of crystallization and adhesive cementation on the fracture resistance of CAD/CAM advanced lithium disilicate and zirconia-reinforced lithium silicate ceramics after thermocycling

**DOI:** 10.3389/fdmed.2026.1715080

**Published:** 2026-02-04

**Authors:** Andrea Ordóñez Balladares, Luis Chauca-Bajaña, Marcela Alejandra Ordoñez Balladares, Rolando Fabricio Dau Villafuerte, Carlos Carpio-Cevallos, César Humberto Palacios Jurado, Elizabeth Cecilia Ortíz Matías, Gina Vasquez Armas, Juan Suarez-Palacios, Byron Velásquez Ron

**Affiliations:** 1 College of Dentistry, University of Guayaquil, Guayas, Ecuador; 2 College of Dentistry, Universidad Bolivariana del Ecuador, Durán, Ecuador; 3 College of Dentistry, Universidad San Gregorio de Portoviejo, Portoviejo, Ecuador; 4 Carrera de Odontología, Department Prosthesis Research, Universidad de Las Américas (UDLA), Quito, Ecuador

**Keywords:** adhesive cementation, advanced lithium disilicate, CAD/CAM ceramics, crystallization, fracture resistance, thermocycling, zirconia-reinforced lithium silicate

## Abstract

**Background:**

Advancements in CAD/CAM glass-ceramics have expanded their use in esthetic dentistry; however, the combined influence of thermal processing and adhesive cementation on their mechanical performance after oral aging remains unclear.

**Aim:**

To assess the effect of crystallization and adhesive cementation on the fracture resistance of CAD/CAM advanced lithium disilicate and zirconia-reinforced lithium silicate ceramics following thermocycling.

**Materials and methods:**

Eighty monolithic crowns (*n* = 10/group) were milled from advanced lithium disilicate (CEREC Tessera™), lithium disilicate (e.max CAD), and zirconia-reinforced lithium silicate (Vita Suprinity®). Specimens were assigned to eight groups based on crystallization and cementation protocols. All crowns underwent 5,000 thermocycles (5 °C–55 °C). Fracture resistance was measured under static compressive loading. Non-parametric tests (Kruskal–Wallis and Mann–Whitney *U* with Bonferroni correction) were applied (*α* = 0.05).

**Results:**

Both crystallization and adhesive cementation significantly increased fracture resistance (*p* < 0.05). Crystallized-cemented specimens exhibited the highest values across all materials. Tessera™ crystallized-cemented crowns reached the highest mean fracture resistance (1,918.9 ± 246.3 N), outperforming e.max CAD and Vita Suprinity®.

**Conclusion:**

Crystallization and adhesive cementation exert synergistic benefits on the fracture resistance of CAD/CAM glass-ceramic crowns after thermocycling. Adhering to recommended thermal cycles and adhesive protocols is essential to optimize mechanical reliability and long-term performance.

## 1 Introduction

The increasing demand for esthetic, metal-free restorations has driven significant advancements in CAD/CAM ceramics. These materials offer favorable optical properties, precision milling, and reliable adhesive bonding, making them suitable for minimally invasive restorations with predictable performance (1–4). Lithium disilicate (LD) and zirconia-reinforced lithium silicate (ZLS) have gained widespread acceptance due to their high flexural strength, improved microstructure, and enhanced fracture toughness compared with earlier glass-ceramic systems (5–7).

Advanced lithium disilicate, such as CEREC Tessera™, incorporates virgilite and lithium-phosphate nanocrystals formed during rapid crystallization, contributing to improved mechanical behavior and accelerated processing protocols (8–10). Crystallization plays a fundamental role in reducing milling-induced flaws and promoting the formation of stable crystalline phases capable of resisting crack propagation (11–13).

Thermal fluctuations and humidity in the oral environment further contribute to degradation of glass-ceramic restorations. Thermocycling is therefore widely used as an accelerated aging protocol to simulate hydrothermal fatigue, with 5,000 cycles often considered the minimum standard for laboratory assessment of early aging effects (14).

Adhesive cementation is another clinical factor critical to the long-term success of ceramic restorations. Through micromechanical interlocking and chemical bonding, resin cements increase load distribution, support thin ceramic margins, and reinforce the ceramic–substrate interface, significantly improving fracture resistance compared with non-adhesive cementation approaches (15–17).

Despite extensive research on crystallization and adhesive cementation independently, limited evidence exists regarding their combined influence on the fracture resistance of advanced LD compared with LD and ZLS after thermocycling. Understanding how these factors interact is essential to optimize clinical protocols and ensure predictable long-term performance of CAD/CAM glass-ceramic restorations.

The aim of this study was to evaluate the influence of heat treatment (crystallization) and adhesive cementation on the fracture resistance of CAD/CAM advanced lithium disilicate and zirconia-reinforced lithium silicate ceramics after thermocycling.

Null hypothesis: Crystallization and adhesive cementation would not significantly affect the fracture resistance of CAD/CAM ceramic crowns after thermocycling.

## 2 Materials and methods

### 2.1 Tooth preparation and digital workflow

**Figure 1:**
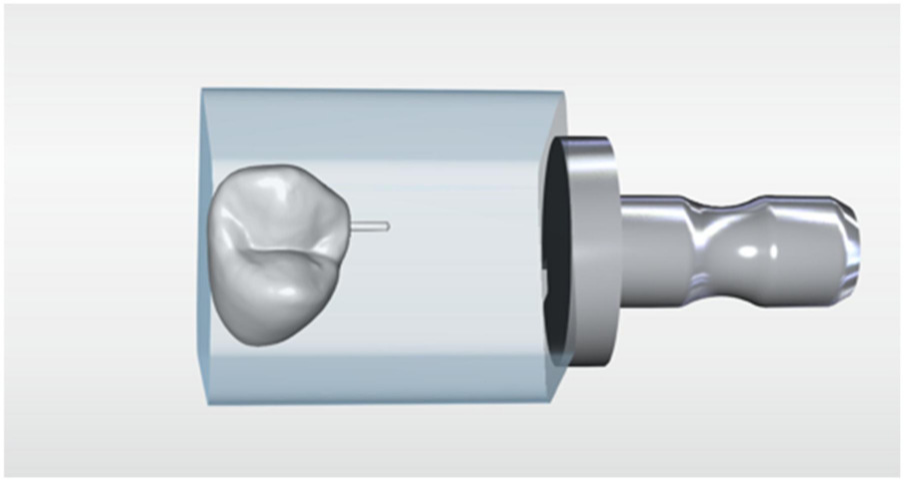
Crown design.

To manufacture the samples, the preparation of a complete crown was used in a model of a first premolar with a 1 mm chamfer-type termination line, the preparation was digitized using a digital intraoral scanner (PrimeScan, Denstply-Sirona™, Germany) as shown in Figure 1. The restoration design was carried out using CAD software (CEREC 5.0, CEREC, Dentsply-Sirona, Germany) and then it was machined with a wet milling machine (CEREC MCXL Premium, Dentsply-Sirona™, Germany), forming 8 groups of 10 samples each as detailed in Table 1.

**Table 1:** Experimental groups.

Group	Material	Composition	Crystallization	Cementation	*n*
G1	Tessera	Li_2_Si_2_O_5_: 90% Li_3_PO_4_:5% Li_0.5_Al_0.5_Si_2.5_O_6_ (virgilite): 5%	No	No	10
G2	Tessera		No	Yes	10
G3	Tessera		Yes	No	10
G4	Tessera		Yes	Yes	10
G5	IPS e.max CAD	SiO_2_: 57%–80% Li_2_O: 11%–19% K_2_O: 0%–13% P_2_O_5_:0%–11% ZrO_2_: 0%–8% ZnO: 0%–8% Coloring oxides: 0%–8%	Yes	No	10
G6	IPS e.max CAD		Yes	Yes	10
G7	Vita Suprinity®	SiO_2_: 56%–64% Li_2_O: 15%–21% ZrO_2_: 8%–12% P_2_O_5_: 3%–8% K_2_O: 1%–4% Al_2_O_3_: 1%–4% CeO_2_: 0%–4% Pigments: 0%–4%	Yes	No	10
G8	Vita Suprinity®		Yes	Yes	10

CEREC Tessera™ was evaluated in four experimental groups because this material is supplied in a pre-crystallized state, allowing assessment of both the non-crystallized and crystallized conditions. In contrast, IPS e.max CAD and Vita Suprinity® require mandatory crystallization as part of their manufacturing protocols; therefore, only crystallized groups were included for these ceramics. The number of groups per material thus reflects the actual processing pathways inherent to each system rather than an experimental imbalance.

### 2.2 Crown fabrication and grouping

Eighty crowns (*n* = 10/group) were milled using a wet-milling unit (CEREC MCXL Premium, Dentsply Sirona™) from three CAD/CAM materials:

Advanced lithium disilicate (CEREC Tessera™)
Lithium disilicate (IPS e.max CAD)
Zirconia-reinforced lithium silicate (Vita Suprinity®)

Minimal post-milling finishing was performed, limited to sprue removal with 600-grit SiC paper under water to avoid introducing surface defects. Final dimensions were standardized:

1.5 mm axial thickness
2.0 mm occlusal thickness

Specimens were distributed into eight groups based on crystallization and cementation status.

CEREC Tessera™ was assessed under both as-milled (no final firing) and crystallized conditions to isolate the biomechanical contribution of the crystallization step. This “non-crystallized” condition was included as an experimental control and does not represent a clinically recommended definitive protocol. In contrast, IPS e.max CAD and Vita Suprinity® require mandatory crystallization according to their manufacturers' processing pathways; therefore, only crystallized groups were included for these materials. Non-cemented groups were included as biomechanical controls to quantify the reinforcing effect of adhesive cementation on fracture resistance under standardized conditions.

### 2.3 Crystallization protocol

Crystallization was performed in a ceramic furnace (Programat P310, Ivoclar Vivadent™, Liechtenstein) strictly following the manufacturers' official Instructions for Use (IFU) for each CAD/CAM ceramic material. The firing protocols complied with the validated factory-recommended parameters, including heating rate, peak temperature, holding time, total cycle duration, and controlled cooling inside the closed chamber.

Peak temperature and holding time for each material are summarized in Table 2. All other thermal parameters were automatically controlled by the furnace software according to the corresponding manufacturer-specific programs to ensure full conformity and reproducibility.

**Table 2:** Heat treatment.

Material name	Ceramic kiln	Maker	Heat units	Time
Cerec Tessera	Programat P310	Ivoclar Vivadent™/Liechtenstein	760 °C	**2 min**
Emax CAD			850 °C	**7 min**
Vita Suprinity			**840 °C**	**8 min**

Bold values represent the manufacturer-recommended crystallization temperature and holding time for each material.

Cooling was completed inside the closed chamber to minimize residual stresses. Non-crystallized groups served as pre-crystallization experimental controls, consistent with mechanical materials science methodologies for isolating the effects of microstructural transformation on fracture behavior.

### 2.4 Composite resin dies

Dies were milled from nanohybrid composite blocks (Grandio® CAD/CAM, VOCO, Germany; modulus of elasticity: 17.1 GPa), selected to approximate dentin stiffness. Each die duplicated the reference preparation and was cleaned with isopropyl alcohol before cementation.

The use of composite resin dies was intended to minimize biological variability and ensure a high level of standardization among specimens. Natural teeth present considerable variations in anatomy, dentin thickness, age-related changes, and substrate properties, which may influence fracture resistance outcomes. The selected composite material provides a reproducible and mechanically consistent substrate with elastic properties comparable to human dentin, allowing controlled stress distribution and improved comparability between experimental groups.

### 2.5 Internal surface conditioning

Internal surfaces of crowns in cementation groups underwent standardized adhesive pretreatment:

Hydrofluoric acid etching
  5% HF gel (Porcelain Etch, Ultradent Products Inc., South Jordan, UT, USA): 20 s (Tessera™ and e.max CAD)
  5% HFgel (Porcelain Etch, Ultradent Products Inc., South Jordan, UT, USA): 60 s (Vita Suprinity®)
  Rinsing: 30 s, followed by 20 s air-drying
Ultrasonic cleaning in distilled water for 3 min using an ultrasonic bath (Elmasonic S 30 H, Elma Schmidbauer GmbH, Singen, Germany)
Silanization
  Silane: Monobond N (Ivoclar Vivadent™, Schaan, Liechtenstein)
  Reaction time: 60 s

Air-abrasion was intentionally avoided to prevent subsurface flaws in brittle ceramic matrices.

### 2.6 Adhesive cementation

Crowns were cemented using a dual-cure adhesive resin cement (Calibra® Ceram, Dentsply Sirona™, Germany). Cementation followed a controlled protocol:

Cement applied to internal crown surface
Seating under 10 N constant load using a calibrated loading device
Removal of excess with microbrushes
Light-curing using Bluephase PowerCure (Ivoclar Vivadent™)
Intensity: 1,200 mW/cm^2^
Distance: 1–2 mm
Duration: 20 s per surface (buccal, palatal, mesial, distal)

Uncemented specimens were intentionally included as biomechanical controls and do not represent a definitive clinical protocol. Their inclusion allowed isolation of adhesive cementation as an independent reinforcing variable, enabling assessment of the structural contribution of resin luting to fracture resistance, while also representing a standardized worst-case scenario that facilitates direct comparison with cemented counterparts under identical geometric and material conditions.

### 2.7 Thermocycling

Thermal aging was performed with a thermocycler (SD Mechatronik, Germany):

5,000 cycles
5 °C/55 °C baths
25 s dwell time
10 s transfer

This protocol is widely adopted for simulating thermal fatigue in preclinical ceramic testing; it does not imply a direct chronological equivalence to clinical years.

### 2.8 Fracture resistance testing

Fracture resistance was assessed using a universal testing machine (Autograph AG-I, Shimadzu, Japan) equipped with a 5 kN load cell:

Crosshead speed: 0.5 mm/min
Loading indenter: 3 mm stainless-steel hemisphere
A thin polyethylene sheet was interposed to avoid localized stress spikes
Load applied along the long axis, centered on the occlusal fossa to contact both cusp slopes
Failure load (*N*) recorded as maximum force prior to catastrophic fracture

### 2.9 Statistical analysis

Sample size determination was based on previously published *in vitro* studies assessing the fracture resistance of CAD/CAM ceramic crowns, in which a sample size of 8–12 specimens per group is commonly used and considered sufficient to detect relevant differences under standardized laboratory conditions. Given the controlled experimental design, the high degree of specimen standardization, and the mechanical nature of the test, a sample size of 10 specimens per group was considered appropriate and consistent with the existing literature.

Analyses were performed in SPSS v27 (IBM Corp., USA). Descriptive statistics included means, medians, standard deviations, coefficients of variation, and 95% confidence intervals.

Normality (Shapiro–Wilk) and homogeneity of variance (Levene) were not satisfied, and data transformations failed to correct deviations. Therefore:

Kruskal–Wallis tests were used for overall comparisons
Mann–Whitney *U* tests with Bonferroni correction were used for pairwise analyses
Statistical significance was set at *p* < 0.05

The inclusion of cemented and non-cemented groups was specifically designed to isolate the reinforcing effect of adhesive luting on fracture resistance within a controlled experimental framework.

Both mean-based and median-based descriptive statistics were reported to provide a comprehensive characterization of fracture resistance data. Means and standard deviations were included to facilitate comparison with previous *in vitro* mechanical studies, where mean fracture loads are commonly reported, whereas medians and interquartile distributions were used for inferential analysis in accordance with the non-parametric statistical approach. All inferential conclusions were therefore based exclusively on rank-based statistics and median-centered comparisons.

## 3 Results

Descriptive statistics are presented using both mean ± standard deviation and median-based measures. Mean values are reported to facilitate comparison with existing fracture resistance literature, whereas all inferential statistical analyses and graphical representations (boxplots) are based on medians and rank-based distributions, in accordance with the non-parametric tests employed.

### 3.1 Descriptive fracture resistance values

A total of 80 crowns were tested. Table 3 summarizes the maximum fracture load for each material–treatment combination. Across all materials, cemented groups demonstrated substantially higher fracture resistance compared with uncemented ones. Uncemented specimens ranged from 468.6 to 754.3 N, whereas cemented groups ranged from 1,481.3 to 1,918.9 N.

**Table 3:** Descriptive summary of the maximum force (*N*) according to material and treatment.

Material/Treatment	Median (*N*)	Mean (*N*)	SD	CV (%)	95% CI
IPS e.max CAD—Crystallized—Uncemented	403.8	468.6	171.0	36.5	(346.8–590.4)
IPS e.max CAD—Crystallized—Cemented	1,373.9	1,509.6	389.0	25.8	(1,387.9–1,631.4)
CEREC Tessera—Crystallized—Uncemented	751.6	754.3	99.4	13.2	(632.6–876.1)
CEREC Tessera—Non-crystallized—Uncemented	477.9	522.9	129.4	24.8	(401.2–644.7)
CEREC Tessera—Crystallized—Cemented	1,929.6	1,918.9	246.3	12.8	(1,797.1–2,040.7)
CEREC Tessera—Non-crystallized—Cemented	1,535.6	1,593.3	230.1	14.4	(1,471.5–1,715.1)
Vita Suprinity—Crystallized—Uncemented	579.9	545.8	211.2	38.7	(424.0–667.6)
Vita Suprinity—Crystallized—Cemented	1,475.2	1,481.3	206.9	14.0	(1,359.5–1,603.0)

**Figure 2:**
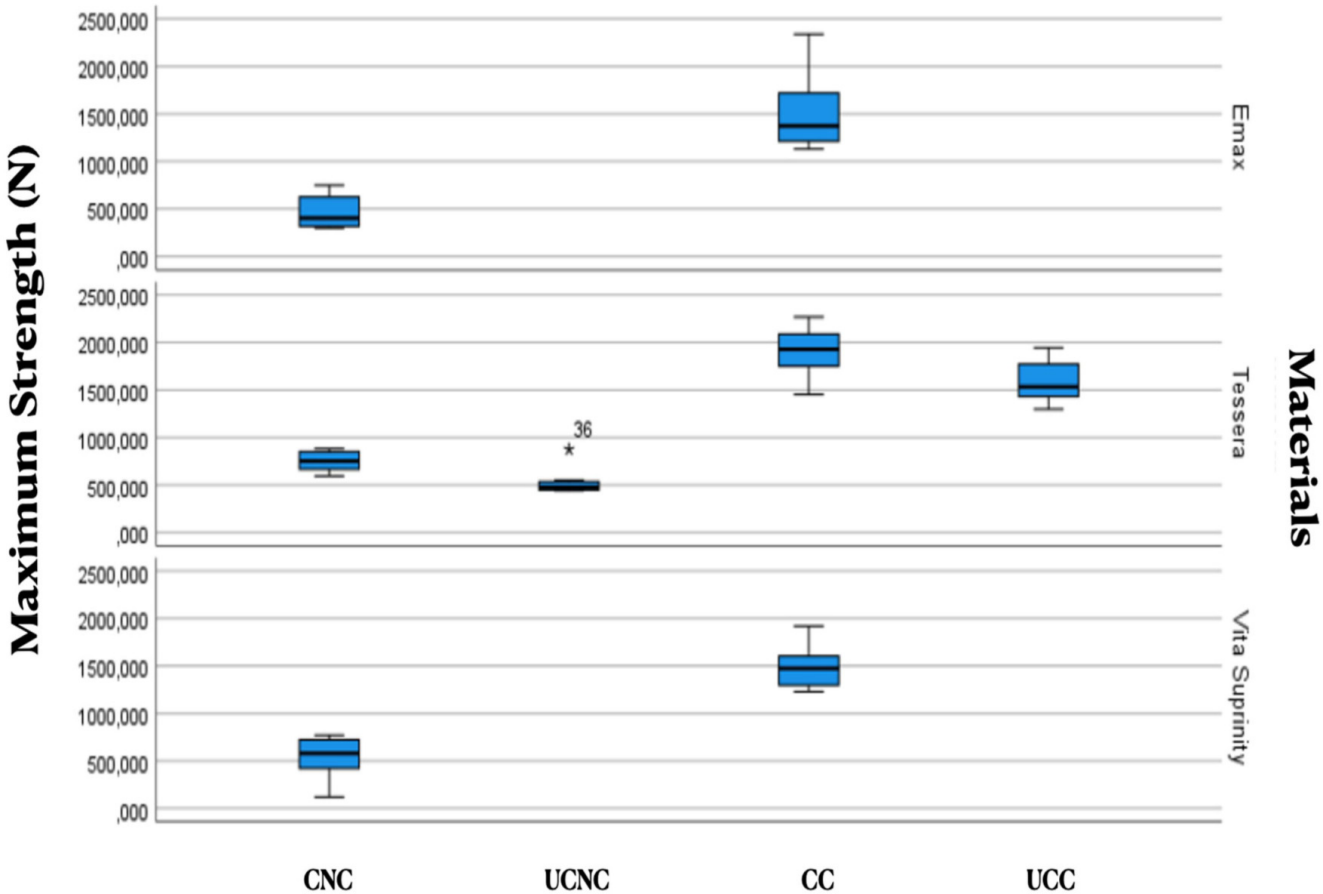
Box plot showing the distribution of fracture resistance values for each treatment combination across the three ceramic materials. Treatment abbreviations are defined as follows: UCNC, uncrystallized–uncemented; CNC, crystallized–uncemented; UCC, uncrystallized–cemented; CC, crystallized–cemented.

Figure 2 shows the distribution of fracture resistance for each treatment combination across the three ceramic materials.

### 3.2 Effect of treatment within each ceramic material

Non-parametric analyses confirmed that treatment type significantly affected fracture resistance within each material:

IPS e.max CAD: *U* = 100, *p* < 0.001
CEREC Tessera: *H* = 33.32, df = 3, *p* < 0.001
Vita Suprinity: *U* = 100, *p* < 0.001

For CEREC Tessera™, crystallized–cemented crowns exhibited significantly higher fracture resistance compared with all untreated or non-crystallized groups (*p* < 0.05). Crystallized–uncemented crowns did not show significant differences when compared with non-crystallized–uncemented specimens (*p* > 0.05). No significant differences were found between crystallized–cemented and non-crystallized–cemented groups after Bonferroni correction (Table 4).

**Table 4:** Pairwise comparisons within CEREC Tessera™.

Comparison	Test statistic	Adjusted *p*-value
Non-crystallized—Uncemented vs. Crystallized—Uncemented	8.2	0.701
Non-crystallized—Uncemented vs. Non-crystallized—Cemented	−20.8	0.000*
Non-crystallized—Uncemented vs. Crystallized—Cemented	−27.4	0.000*
Crystallized—Uncemented vs. Non-crystallized—Cemented	−12.6	0.096
Crystallized—Uncemented vs. Crystallized—Cemented	−19.2	0.001*
Non-crystallized—Cemented vs. Crystallized—Cemented	6.6	1.000

Negative values represent the directionality of the Mann–Whitney *U* test statistic.

*Indicates a statistically significant difference after adjustment for multiple comparisons (*p* < 0.05).

### 3.3 Comparison among ceramic materials

When all treatments were pooled within each ceramic, the Kruskal–Wallis test showed no significant difference in fracture resistance among the three materials (*H* = 3.393; df = 2; *p* = 0.183). Median fracture resistance values were: IPS e.max CAD = 941.0 N, CEREC Tessera = 1,090.4 N, and Vita Suprinity = 1,001.6 N (Table 5).

**Table 5:** Fracture resistance according to ceramic material.

Material	Median (*N*)	Mean (*N*)	SD	CV (%)	95% CI
IPS e.max CAD	941.0	989.1	608.9	61.6	704.2–1,274.1
CEREC Tessera	1,090.4	1,197.4	610.7	51.0	1,002.0–1,392.7
Vita Suprinity	1,001.6	1,013.5	521.2	51.4	769.6–1,257.5

**Figure 3:**
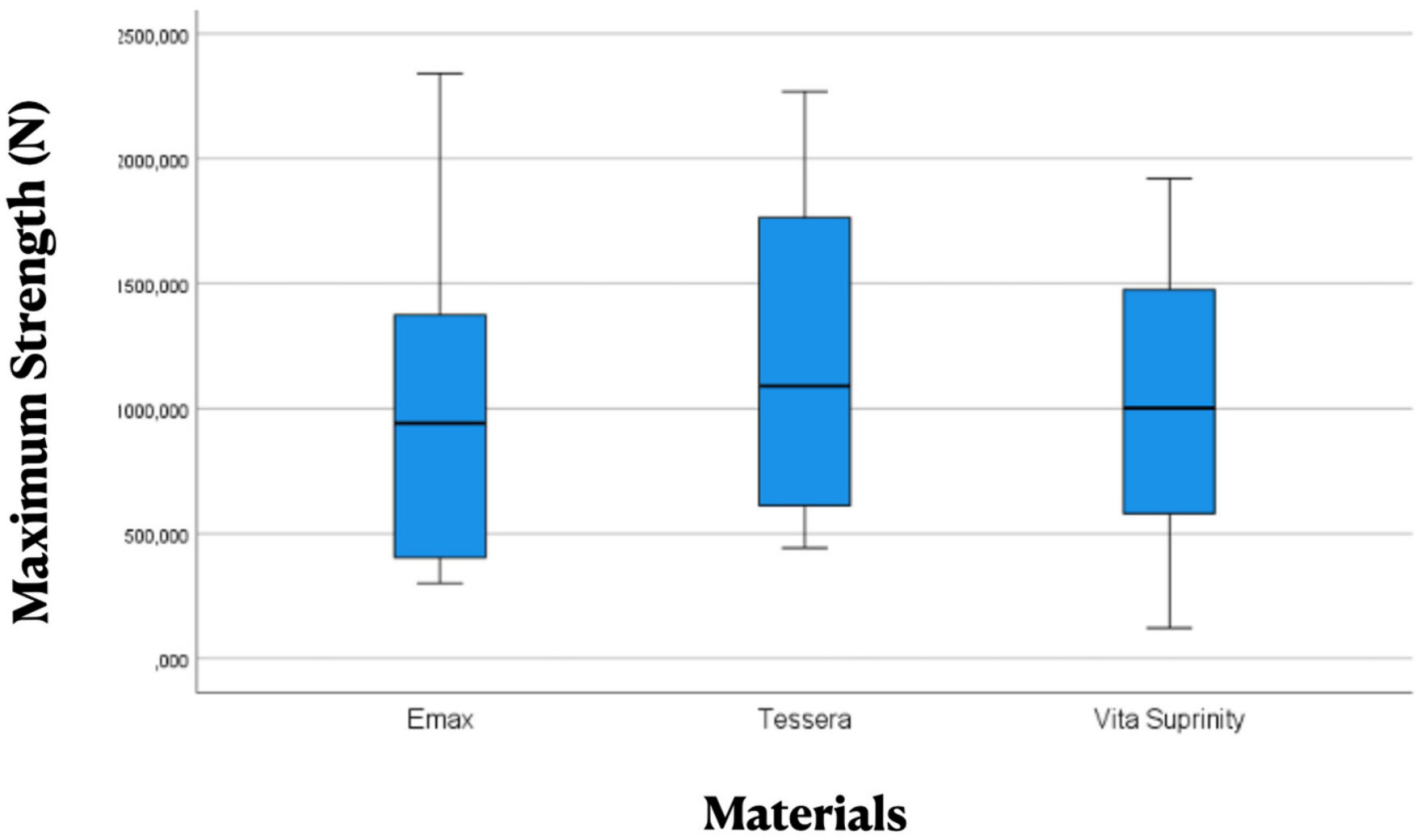
Box plot comparing fracture resistance values among the three ceramic materials. Data include all treatment conditions. Treatment abbreviations are defined as follows: UCNC, uncrystallized–uncemented; CNC, crystallized–uncemented; UCC, uncrystallized–cemented; CC, crystallized–cemented.

Figure 3 shows the distribution of fracture resistance values for each ceramic material, illustrating the median, interquartile range, and overall variability. Although CEREC Tessera™ exhibited the highest median fracture resistance, followed by Vita Suprinity® and IPS e.max CAD, considerable overlap of the interquartile ranges was observed among the three materials, which is consistent with the absence of statistically significant differences.

### 3.4 Effect of treatment type across materials

When treatment types were analyzed irrespective of ceramic material, significant differences in fracture resistance were observed (*H* = 59.579; df = 3; *p* < 0.001; Table 6). Non-crystallized–uncemented crowns showed significantly lower fracture loads than all other treatment groups (*p* < 0.001). Crystallized–uncemented crowns differed significantly from both cemented treatments (*p* < 0.001). No significant difference was detected between crystallized–cemented and non-crystallized–cemented crowns after Bonferroni adjustment.

**Table 6:** Overall treatment effect across materials.

Treatment	Median (*N*)	Mean (*N*)
Non-crystallized—Uncemented	492.0	509.1
Crystallized—Uncemented	679.3	682.9
Non-crystallized—Cemented	1,458.2	1,507.0
Crystallized—Cemented	1,628.5	1,680.7

Kruskal–Wallis: *H* = 59.579, df = 3, *p* < 0.001. Pairwise *post hoc* (Bonferroni): all comparisons were statistically significant except Non-crystallized—Cemented vs. Crystallized—Cemented (*p* = 0.252).

## 4 Discussion

The present study analyzed the independent and combined influence of crystallization and adhesive cementation on the fracture resistance of CAD/CAM lithium-disilicate–based ceramics after thermocycling. Overall, both factors significantly increased fracture resistance, and their combined application produced the highest fracture resistance values under crystallized-and-cemented conditions. These findings are aligned with the scientific consensus that mechanical performance of glass-ceramics is strongly dependent on microstructural development, surface integrity, and bonding reinforcement (18–27).

Crystallization significantly improved fracture resistance in CEREC Tessera™, the only material in which crystallization was experimentally manipulated in this study. This finding is consistent with previous evidence indicating that heat treatment promotes crystal growth, reduces internal defects, and enhances crack-deflection and crack-bridging mechanisms in lithium disilicate–based and zirconia-reinforced lithium silicate ceramics, as reported in the literature (28–34).

During crystallization, the transition from glassy matrices to well-defined crystalline structures decreases residual stresses and improves the homogeneity of the microstructure. Studies have demonstrated that proper firing eliminates or neutralizes milling-induced flaws, increases flexural strength, and enhances resistance to slow crack growth under thermal and mechanical stress (35–42).

Advanced LD systems such as CEREC Tessera™ benefit from virgilite and lithium-phosphate nanocrystals, which form during rapid crystallization cycles and significantly enhance fatigue behavior and fracture toughness (43–48). The rapid-crystallization strategy produces distinctive crystalline arrangements that are absent in conventional LD, contributing to favorable short-term mechanical performance after processing (49–51).

Mismanagement of firing parameters may compromise ceramic performance. Excessive firing may increase brittleness, while insufficient crystallization may lead to incomplete phase development, increased flaw sensitivity, and lower strength (52–54). Such findings underscore the importance of adhering strictly to manufacturer-specified thermal cycles to ensure predictable and optimal mechanical outcomes.

Adhesive cementation also played a major role in reinforcing ceramic crowns. Resin-based luting agents enhance fracture resistance by improving stress distribution, providing internal support, and strengthening the ceramic–substrate interface (55–58).

Surface conditioning procedures—such as hydrofluoric acid etching, silanization, or the use of MDP-containing primers—promote durable micromechanical and chemical adhesion between ceramic and cement, reducing interfacial defects and increasing fracture loads (59–63).

Bond durability is influenced by dentin moisture, adhesive chemistry, and the resilience of the cement itself. Several studies show that the adhesive interface is often the most vulnerable region to hydrothermal degradation, making proper bonding techniques essential for long-term performance, especially after aging protocols such as thermocycling (64–66).

These observations explain why cemented specimens in the present study demonstrated significantly higher fracture resistance compared with non-cemented controls, corroborating earlier findings that adhesive luting converts brittle ceramic behavior into a reinforced, stress-modulated system (67–70).

The highest fracture resistance values were achieved in crystallized-and-cemented specimens across all evaluated materials, confirming a synergistic interaction between optimized microstructure and adhesive reinforcement. Crystallization enhances the ceramic's resistance to crack initiation, while adhesive cementation reduces tensile stress concentrations at critical areas and stabilizes the ceramic during loading (43–48, 67–70).

This synergy has been previously observed in CAD/CAM LD, ZLS, and advanced LD systems, where the combination of proper firing and resin bonding consistently yields superior mechanical results compared with either strategy used alone (71–73).

Notably, although cementation improved the non-crystallized crowns, the absence of full microstructural development likely limited their strength compared with crystallized specimens. This reinforces the clinical relevance of crystallization as an irreplaceable step.

Despite numerical differences among materials, no statistically significant differences were observed once crystallization and adhesive cementation were applied. This indicates that CAD/CAM LD, ZLS, and advanced LD can reach similar fracture thresholds under standardized geometry, preparation thickness, and bonding conditions (1–7, 38–43).

Comparable behaviors among these ceramics have been reported in evaluations of fatigue resistance, flexural strength, interface integrity, and surface roughness after milling and aging (44, 45, 58–61). Therefore, material selection may prioritize optical performance, translucency requirements, clinical indication, and processing characteristics, rather than mechanical superiority alone.

Thermocycling induces hydrothermal stresses that mimic early intraoral aging by exposing materials to temperature gradients and moisture penetration. Although 5,000 cycles represent an early aging stage, the protocol is well established to reveal vulnerabilities related to hydrothermal degradation, interfacial breakdown, and crack propagation (35–41).

In this study, crystallized–and-cemented specimens maintained higher strength after thermocycling, reinforcing the importance of both processes for resisting hydrothermal fatigue. However, thermocycling alone does not replicate full oral complexity, which also includes pH changes, enzymatic degradation, cyclic masticatory loading, and biofilm activity—factors requiring long-term combined aging protocols for future research.

A major strength of this study lies in its rigorous standardization of tooth preparation, ceramic thickness, milling pathways, surface conditioning, cementation protocols, and mechanical testing. This controlled environment minimizes confounders and increases the reliability of observed differences.

However, limitations include the use of static load testing rather than cyclic fatigue, the absence of microstructural characterization (SEM/XRD) following different firing cycles, and the short-term nature of thermocycling. Future investigations should incorporate long-term fatigue, advanced imaging, and multi-parametric aging to better approximate clinical conditions and validate these findings.

## 5 Conclusion

The combined application of crystallization and adhesive cementation produced the highest fracture resistance across all CAD/CAM glass-ceramic materials evaluated. Both treatments independently improved mechanical behavior, but their synergistic effect yielded superior strength and lower variability. Among the tested materials, advanced lithium disilicate (CEREC Tessera™) achieved the highest fracture resistance under standardized conditions after thermocycling. These results underscore the clinical importance of adhering to manufacturer-recommended crystallization cycles and applying adhesive luting protocols to optimize the structural reliability and long-term success of ceramic restorations.

## Data Availability

The original contributions presented in the study are included in the article/Supplementary Material, further inquiries can be directed to the corresponding author.
